# Hepatitis A beyond childhood causing diagnostic and therapeutic challenges in Addis Ababa, Ethiopia

**DOI:** 10.1371/journal.pone.0330212

**Published:** 2025-08-13

**Authors:** Abate Bane Shewaye, Kaleb Assefa Berhane, Amsalework Daniel Fanta

**Affiliations:** 1 Department of Internal Medicine, College of Health Sciences, Addis Ababa University, Addis Ababa, Ethiopia; 2 Department of Internal Medicine, Adera Medical and Surgical Center, Addis Ababa, Ethiopia; Centers for Disease Control and Prevention, UNITED STATES OF AMERICA

## Abstract

**Background:**

Hepatitis A is an acute viral infection of the liver caused by hepatitis A virus (HAV) that is acquired through the feco-oral route. It has the highest incidence among the four major acute viral hepatitis types (A, B, C, and E) and usually occurs in early childhood. However, the prevalence of acute hepatitis A has recently increased among teenagers and young adults, and it is usually misdiagnosed. This study emphasizes the significance of awareness among healthcare workers about the increasing incidence of acute hepatitis A among this group to ensure accurate diagnosis and appropriate management.

**Methods:**

A hospital-based retrospective cross-sectional study was employed. Fifty-eight confirmed acute HAV patients who visited Adera Medical and Surgical Center (AMSC) between August 2023 and January 2024 were enrolled. Sociodemographic, clinical, and laboratory parameters and documented management data, including hospitalization and any trial of antibiotic treatment before considering HAV or in the course of the illness, were collected. The data were entered and analyzed using SPSS (SPSS, Version 26.0).

**Results:**

The sex ratio was similar, with a slight male predominance (M/F = 1.07). The mean age [± SD] of the patients was 19.3[± 8.8] years. Thirty-nine (67.2%) of the patients were students, and all of the patients were from Addis Ababa. Vomiting (82.8%), anorexia (70.7%) and yellowish discoloration of the eyes (62.1%) were the most common presenting symptoms, while icteric sclera 44 (75.9%) and epigastric tenderness 17 (29.3%) were the most common physical findings. More than half of the patients (55.2%) were initially misdiagnosed with typhoid fever (TF) (46.8%), peptic ulcer disease (PUD) (31.2%) or urinary tract infection (UTI) (15.6%). All patients recovered fully, and liver function tests (LFTs) normalized with supportive care within 2–4 weeks.

**Conclusion:**

This study revealed the shift in the age of HAV susceptibility and subsequent infection towards adolescents and young adults (mean [± SD] age 19.31 [± 8.8] years) in our cohort, with more than half of the patients (55.2%) initially being misdiagnosed with TF, PUD or UTI, causing diagnostic and treatment challenges. This necessitates heightened awareness among healthcare workers and the public. Early HAV diagnosis through targeted laboratory investigations and avoiding unnecessary antibiotics are crucial for effective management and prevention via hygienic and immunization strategies.

## Introduction

Hepatitis A is an acute inflammation of the liver caused by hepatitis A virus (HAV), a single-stranded RNA virus of the Picornaviridae family. It is a vaccine-preventable disease that mainly spreads through the feco-oral route either by direct close contact with an HAV-infected person or by ingestion of food or water contaminated by the feces of an infected person [1–3].

HAV is endemic in low- to middle-income countries due to poor sanitary conditions, where most children are infected with hepatitis A before the age of 10. The World Health Organization estimates that approximately 1.5 million people are infected with HAV annually worldwide, resulting in 15,000–30,000 deaths per year. However, improving sanitary conditions in these countries has shifted the incidence of HAV to adolescents and young adults, the age at which the immune system strongly reacts to the infection and results in more severe disease symptoms [4–6].

The clinical presentation of HAV can range from asymptomatic to fulminant liver failure. The manifestation and severity of symptoms after HAV infection directly correlate with age. It is usually asymptomatic in children, and older patients are at increased risk of severe disease [6,7]. The incubation period of HAV lasts 15–45 days, followed by a nonspecific prodromal phase, with malaise, fever, and anorexia being the most common symptoms. Vomiting, diarrhea, and abdominal discomfort may also be present. The icteric phase manifests with jaundice and hepatic cytolysis with elevated LFTs. Hepatomegaly and gallbladder wall thickening can be found on sonography [1,6,8]. HAV diagnosis is confirmed by the presence of anti-HAV-IgM autoantibodies, which appear 5–10 days before the onset of symptoms and may persist for 4–6 months.

Hepatitis A is a self-limiting illness that usually requires supportive management with intravenous fluid resuscitation, electrolyte correction, and anti-emetics. In most cases, HAV resolves completely on its own. Hospitalization is indicated if there is severe intractable vomiting, severe electrolyte and fluid imbalance, or altered mental status. The major cause of death in patients with HAV infection is fulminant hepatic failure, which occurs in <1% of patients [2,6]. Relapse of HAV can occur in 3–20% of patients, usually 3–12 weeks after initial presentation, but symptoms are less severe [6].

Once primarily a childhood illness, hepatitis A, is exhibiting a concerning shift in demographics. Due to improved sanitation and hygiene practices, exposure to the virus is often delayed, leading to a surge in infections among teenagers and young adults with outdoor exposure in Ethiopia [9]. The nonspecific nature of initial symptoms can mimic other conditions, leading to confusion and missed diagnoses. Moreover, healthcare providers may not be sufficiently aware of the increasing prevalence of hepatitis A in young adults, further contributing to the diagnostic dilemma. The objective of this study was to assess the pattern of clinical hepatitis A infection diagnostic and management practices in Addis Ababa, Ethiopia.

## Materials and methods

This retrospective cross-sectional study was conducted to assess the clinical presentation, diagnostic practices and management trends of HAV patients from August 2023 to January 2024. The study took place at Adera Medical and Surgical Center (AMSC), a renowned dedicated gastrointestinal and hepatology center that offers specialized care to thousands of patients in Addis Ababa and regions of Ethiopia.

All patients who came to AMSC gastrointestinal and hepatology unit with clinical symptoms and laboratory findings consistent with acute hepatitis A were included in the study, and patients considered to have acute hepatitis but not confirmed with laboratory investigations, positive autoantibody tests or missing data were excluded.

The diagnosis of HAV was confirmed by the detection of anti-HAV IgM antibodies using enzyme-linked immunosorbent assay (ELISA) kit. Blood samples were collected from patients suspected of HAV infection, and the samples were tested for anti-HAV IgM antibodies using an ELISA kit following the manufacturer’s protocol. Serum was separated from whole blood, and the test was performed according to standard ELISA procedures. Positive results were confirmed based on the optical density values exceeding the cutoff recommended by the kit manufacturer.

Data for this study were extracted from the electronic medical records system between 19/02/2024, and 28/02/2024, by two of the authors using a pretested data abstraction format that was prepared by reviewing relevant works of literature. Confidentiality of individual patient information was maintained by using code numbers instead of other identifiers, and the information obtained from the chart was used solely for research purposes.

A total of 91 patients presenting with clinical symptoms of acute hepatitis were recruited for the study during the study period. Of these, 14 patients were excluded due to missing data, 10 were excluded due to positive autoantibody tests for autoimmune hepatitis, and 9 were excluded due to the lack of laboratory confirmation of HAV. Consequently, 58 patients were included in the final analysis. Data were collected by reviewing the patients’ medical records. The acquired data were examined for accuracy, cleaned, and validated before analysis using SPSS 26.0. For categorical variables, descriptive statistics were utilized as a statistical data analysis technique and are expressed as frequencies and numbers (percentages). Tables and figures were used to summarize the results. Categorical data are represented as frequencies, whereas continuous variables are represented as the means, standard deviations, and minimum and maximum values.

## Results

The mean (± SD) age of the patients was 19.31 [± 8.8] years. Approximately two-thirds (63.7%) of the patients were older than 15 years, with ages ranging between 4 and 37 years. Thirty (51.7%) of them were males, while 28 (48.3%) were females. All of the patients were urban dwellers residing in Addis Ababa, 39 (67.2%) of whom were students (Table 1).

**Table 1:** Sociodemographic characteristics of HAV patients visiting AMSC, August 2023 to January 2024.

Characteristics		Total (n = 58)	
		Frequency	Percent (%)
Gender	Male	30	51.7
	Female	28	48.3
Age groups	0 to 5 years	2	3.4
	6 to 10 years	11	19.0
	11 to 15 years	8	13.8
	16 to 20 years	10	17.2
	21 to 25 years	10	17.2
	Above 25 years	17	29.3
Address	Addis Ababa	58	100
Occupation	Student	39	67.2
	Health care worker	1	1.7
	Teacher	1	1.7
	Businessman	6	10.3
	Accountant	7	12.0
	Government officer	1	1.7
	Unemployed	3	5.2

**Fig 1:**
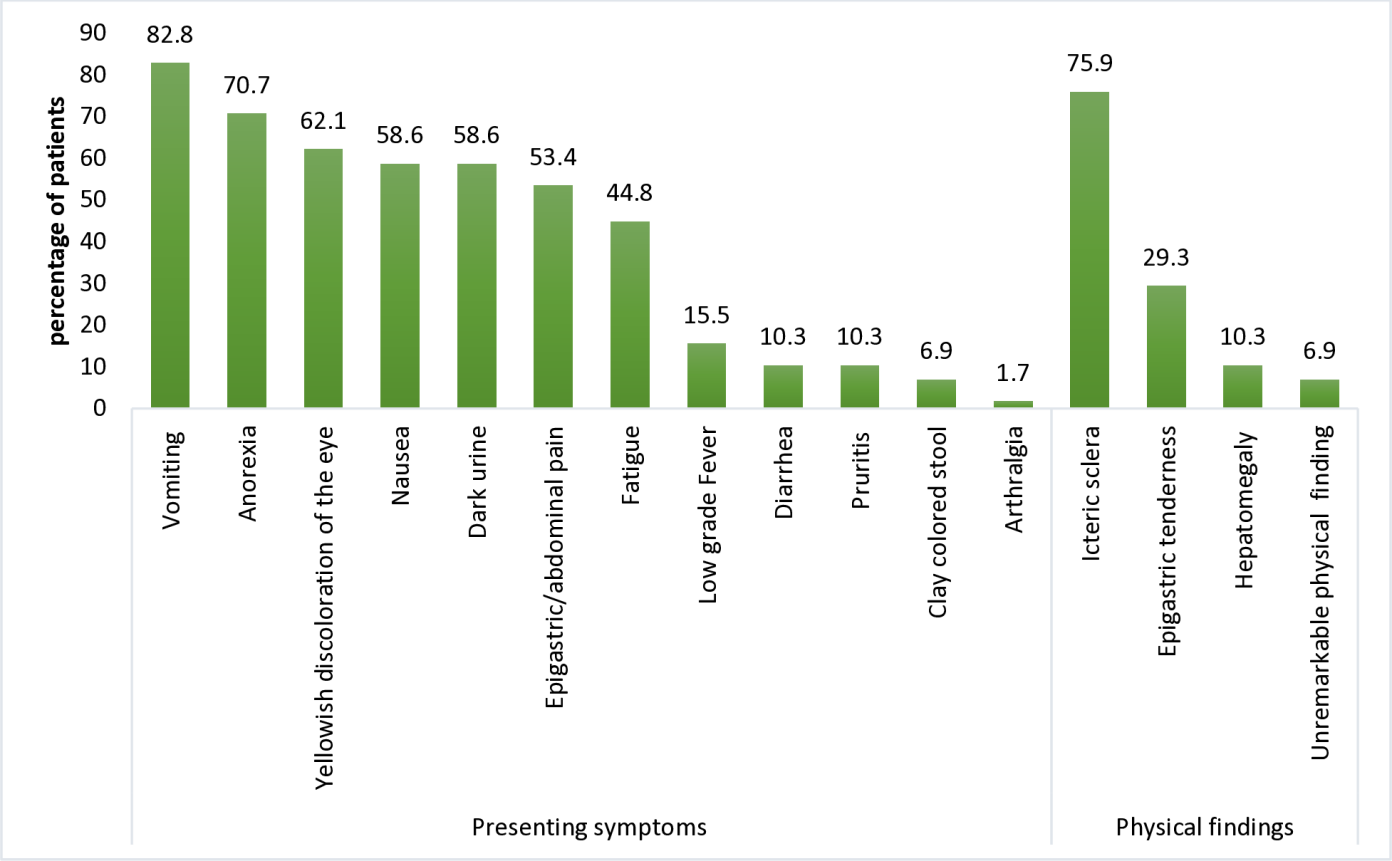
Clinical Presentation of HAV patients visiting AMSC, August 2023 to January 2024.

The median duration of illness before presentation to AMSC was seven days (range 2–30 days). The most common presenting symptoms were vomiting (82.8%), anorexia (70.7%), and yellowing of the eyes (62.1%). Only 1 patient reported arthralgia. Icteric sclera was the most common physical finding in 44 patients (75.9%), followed by epigastric tenderness in 17 patients (29.3%) (Fig 1).

Two patients presented with extrahepatic manifestations (acute cholecystitis), and only 6 (10.5%) of the patients had a history of contact with a jaundiced person, while risk (lack of safe water, use of recreational drugs, raw meat consumption, contact with someone with acute hepatitis A infection, living in a household with an infected person) was not identified or documented in 44 (75.9%) and 8 (13.8%) of the patients, respectively. All patients did not have history of vaccination for HAV.

Laboratory investigation revealed significant increases in AST, ALT, and total and direct bilirubin, while ALP was less than two and a half times greater than the upper normal limit in the majority of the patients. Leukocytosis was detected in four patients. Hemoglobin and platelet results were normal in 87.9% of the patients. All of the patients tested positive for anti-HAV IgM but were negative for HBsAg, HCVab and HIV tests.

Sixty-two percent of the patients had hepatomegaly on ultrasonographic abdominal examination, while thickened gallbladder wall and splenomegaly were observed in 29.3% and 3.4% of the patients, respectively. Fatty liver, hepatic hemangioma and bright liver were reported in 8.6% of the patients (Table 2).

**Table 2:** Laboratory and imaging findings of HAV patients visiting AMSC, August 2023 to January 2024.

Variables		Total, n = 58	
		Frequency	Percent (%)
**Laboratory investigations**			
WBC count (in/L)	≤ 4000	16	27.6
	4000–11,000	38	65.5
	>11,000	4	6.9
Hemoglobin level (in g/dl)	Severe Anemia (≤ 7)	0	0
	Moderate Anemia (>7.0–10.0)	2	3.4
	Mild Anemia (>10–12.5)	4	8.6
	Normal (≥ 12.5)	51	87.9
Platelet count (in/L)	Thrombocytopenia (<150 x 10^3^)	6	10.3
	Normal (150 x 10^3^–450 x 10^3^)	51	87.9
	Thrombocytosis (>450 x 10^3^)	1	1.7
ALT value (in IU/L)	Normal (≤40)	0	–
	below 2.5 times elevated	5	8.6
	2.5 to 5 times elevated	2	3.4
	More than 5 times elevated	51	87.9
AST value (in IU/L)	Normal (≤ 40)	1	1.7
	below 2.5 times elevated	4	6.9
	2.5 to 5 times elevated	14	24.1
	More than 5 times elevated	39	67.2
ALP value (in IU/L)	Normal (≤ 147)	2	3.4
	below 2.5 times elevated	31	53.4
	2.5 to 5 times elevated	19	32.8
	More than 5 times elevated	6	10.3
GGT value (in IU/L), n = 47	Normal (≤ 30)	2	4.3
	below 2.5 times elevated	5	10.6
	2.5 to 5 times elevated	17	36.2
	More than 5 times elevated	23	48.9
Direct Bilirubin (in mg/dl), n = 57	Normal (<0.3)	2	3.5
	below 2.5 times elevated	6	10.5
	2.5 to 5 times elevated	10	17.5
	More than 5 times elevated	39	68.4
Total Bilirubin, in mg/dl	Normal (≤ 1.2)	6	10.3
	below 2.5 times elevated	7	12.1
	2.5 to 5 times elevated	23	39.5
	More than 5 times elevated	22	37.9
**Imaging**			
Abdominal ultrasound exam findings, n = 58	Hepatomegaly	36	62.1
	Thickened gall bladder wall	17	29.3
	Splenomegaly	2	3.4
	Fatty liver	3	5.1
	Hepatic hemangioma	1	1.7
	Bright Liver	1	1.7

More than half of the patients (55.2%) visited other medical centers before presenting to AMSC, and TF (43.8%), PUD (31.2%) and UTI (15.6%) were considered as initial diagnoses. Autoimmune hepatitis and gallstone disease were also diagnosed in four of the patients. Ciprofloxacillin (43.3%), PPIs (33%) and doxycycline (9.9%) were the most commonly prescribed drugs for the patients. Antibiotics such as cotrimoxazole, ceftriaxone, and cefixime were also given to 6 (20%) patients, while one patient received unspecified medication before presenting to our center (Table 3).

**Table 3:** Initial Clinical diagnosis and treatment history of HAV patients visiting AMSC, August 2023 to January 2024.

Variables		Total, n = 58	
		Frequency	Percent (%)
Previous visit to another hospital/clinic	Yes	32	55.2
	No	22	37.9
	Unknown	4	6.9
Diagnosis during previous visit to hospital/clinic, n = 32	TF	14	43.8
	UTI	5	15.6
	PUD	10	31.3
	Autoimmune hepatitis	2	6.25
	Gall stone disease	2	6.25
	Unspecified	2	6.25
Was medication prescribed during previous visit to another hospital/clinic, n = 32	Yes	30	93.75
	No	2	6.25
Prescribed medications during the previous hospital/clinic visit, n = 30	Ciprofloxacillin	13	43.3
	Doxycycline	3	9.9
	Cotrimoxazole	2	6.6
	PPI	10	33.3
	Others(ceftriaxone,cefixim)	4	13.3
	Unspecified	1	3.3

Eleven (19%) patients with severe electrolyte and fluid imbalance required hospitalization at our center during their management with average hospital stay of 2.9 days before being discharged with clinical and biochemical improvements. The patients who were diagnosed with acute HAV in our center were treated with intravenous fluid (for admitted patients), multivitamin, Livolin (phosphatidylcholine 300 mg + B vitamins + vitamin E) and dietary advice and recovered fully within 2–4 weeks with normalized LFTs. No complications or deaths occurred in our HAV cohort.

## Discussion

Hepatitis A is an enteric virus infection that is closely associated with socioeconomic status, poor sanitary conditions, and hygiene practices, leading to infection during early childhood, at which age the immune system is immature and the immune reaction to the infection is weak, resulting in no or mild symptoms and lasting acquired immunity [2,4,9].

With socioeconomic status growth and improvements in hygiene and access to clean water, the acquisition of hepatitis A has shifted to children, adolescents and young adults with more outdoor exposure to contaminated water and food. At this age, the disease will be severe and complicated as damage to hepatocytes is caused by the reaction of the mature strong immune system more than the virus itself. [2,9,10].

Children, adolescents, and young adults from high-income families acquire Hepatitis A when they are exposed to the virus for the first time through food or drink contaminated with HAV at schools, restaurants, or colleges after being brought up in a relatively clean environment. The host immune reaction to HAV at this age is aggressive, resulting in severe hepatitis and manifestations, in contrast to HAV infection during early infancy in poor economic lifestyles [9,11].

A study done in Brazil and Mexico revealed that the mean age of symptomatic HAV patients has shifted from childhood to early adulthood as safe water access has improved [12]. A similar pattern of a shift in the endemicity of hepatitis A was also shown in a study conducted in India in 2019; as the majority of the population is no longer exposed to HAV in childhood, the disease remains highly endemic, resulting in more severe disease and outbreaks in older and susceptible individuals [13]. Studies from other developing countries in Africa, Asia, and Latin America have also shown similar patterns [14–16].

An age-specific seroprevalence study conducted in Ethiopia revealed a 50% HAV antibody seroprevalence among children, while almost all individuals were positive for anti-HAV antibody by the age of 15 [17]. In contrast, our study showed that clinical acute hepatitis A is becoming common among adolescents and young adults from urban settings, as approximately two-thirds (63.7%) of the patients were above the age of 15 years, and all of the patients lived in the capital city, Addis Ababa. This is more than what was reported by a study calling for the inclusion of HAV vaccine in the National Expanded Program on Immunization (EPI) program, which was conducted at the same center, AMSC, in Addis Ababa, Ethiopia, in 2021, and showed 48% of patients with Hepatitis A to be above the age of 15 years and 89% of patients were from high-income and middle-income families [9].

Given the potential for person-to-person transmission, particularly through shared food or close contact, post-exposure prophylaxis (PEP) plays a critical role in preventing secondary cases of hepatitis A [18]. Although no symptomatic illness was reported among close contacts of the patients included in our study at the time of presentation, international guidelines recommend administration of immune globulin or a single dose of hepatitis A vaccine within two weeks of exposure to reduce the risk of infection among contacts. However, hepatitis A vaccine is currently not part of Ethiopia’s national immunization program and is generally unavailable in public or private facilities. In light of the shifting epidemiology of HAV in urban areas, consideration should be given to the potential public health benefits of introducing targeted hepatitis A vaccination strategies. A single dose of the vaccine has been shown to induce protective antibody levels in nearly all recipients within 2–4 weeks [18], and could serve as an effective tool for both outbreak control and long-term prevention in settings with improving sanitation.

The shifting epidemiology of HAV in Ethiopia presents new challenges for diagnosis and treatment. The nonspecific nature of initial symptoms and health care providers having a low index of suspicion for hepatitis A in young adults with acute hepatitis has led to misdiagnosis and treatment.

Misdiagnosis can have significant implications for public health, including delayed diagnosis, increased risk for transmission in the community and outbreaks. Moreover, unnecessary tests, interventions, and treatments drain financial resources and burden patients and the healthcare system.

The use of antibiotics such as ciprofloxacin, doxycycline, and cotrimoxazole—prescribed to nearly one-third of patients (31%)—poses a risk of drug-induced liver injury. This may further confound the clinical picture of hepatitis and contribute to the development of acute liver failure, a condition associated with significant morbidity and mortality.

## Conclusion

This study revealed an epidemiological shift in acute HAV from childhood to adolescence and young adulthood in an urban setting cohort of patients. More than half of the patients were initially misdiagnosed with TF, PUD or UTI. Clinicians need to be especially aware of this shift in hepatitis A among adolescent and young adult patients who initially present with prodromal acute hepatitis. This will help avoid empirical hepatotoxic antibiotics, effective treatment, prevention of local outbreaks, and health cost savings. We recommend conducting national HAV seroprevalence surveys and evaluating current hepatitis A control strategies to inform targeted public health interventions.
